# Synovial Fluid MicroRNA-210 as a Potential Biomarker for Early Prediction of Osteoarthritis

**DOI:** 10.1155/2019/7165406

**Published:** 2019-07-29

**Authors:** Wen Xie, Wei Su, Hualing Xia, Zhanchao Wang, Chunxia Su, Bing Su

**Affiliations:** ^1^Orthopedic Institute of Henan Province, Luoyang, Henan 471002, China; ^2^Department of Orthopedics, The Third Affiliated Hospital, Xinxiang Medical University, Xinxiang, Henan 453003, China; ^3^Luoyang Orthopedic Hospital of Henan Province, Luoyang, Henan 471002, China; ^4^Xinxiang Key Lab of Translational Cancer Research, The Third Affiliated Hospital, Xinxiang Medical University, Xinxiang, Henan 453003, China

## Abstract

Early detection and treatment are critical in the management of osteoarthritis (OA). OA is closely associated with angiogenesis and the inhibition of angiogenesis presents a novel therapeutic approach to reduce inflammation and pain in OA. Recent reports suggest that circulating microRNAs (miRNAs) have great potential as biomarkers for the diagnosis and prognosis in OA. In this study, we aimed to explore the clinical significance of miR-210 in synovial fluid samples from 10 healthy volunteers and 20 early-stage OA and 20 late-stage OA patients. miR-210 expression was assessed by real-time RT-PCR. VEGF protein levels were examined by ELISA. The results show that miR-210 is significantly upregulated in early-stage OA and late-stage OA patients compared with healthy individuals. Higher levels of VEGF are also found in OA compared with the control. Moreover, miR-210 levels are positively correlated with VEGF levels, suggesting that miR-210 might contribute to OA development through promoting VEGF expression and angiogenesis. In conclusion, upregulation of miR-210 in synovial fluid may occur in the early stage of OA and can be a useful biomarker for early diagnosis of OA.

## 1. Introduction

Osteoarthritis (OA), the most common form of arthritis and a major cause of disability, is becoming a major public health problem considering the increasing life expectancy of the population and posing a significant socioeconomic burden worldwide [1]. To date, molecular mechanisms involved in OA remain unclear and a definitive cure is still not available. Therefore, it is urgent for discovery of new biomarkers to facilitate the development of tailored preventive and therapeutic approaches.

Angiogenesis is one of the earliest histopathologic findings in most common forms of chronic, noninfectious arthritis, rheumatoid arthritis (RA) and osteoarthritis (OA) [2]. Neovascularization can maintain chronic inflammatory status by transporting inflammatory cells to the site of synovitis as well as supplying nutrients and oxygen to pannus [3]. Anti-vascular endothelial cell growth factor (VEGF) antibody markedly attenuated disease severity in arthritis [4], indicating that inhibition of angiogenesis may be a novel strategy to OA treatment [5].

Many microRNAs (miRNAs) have been reported to play key roles in gene regulation and contribute to OA pathogenesis [6]. miRNAs not only exist intracellularly, but can be released into almost all body fluids [7]. More importantly, extracellular miRNAs or circulating miRNAs are sensitive, easily detectable and highly stable [8], suggesting that miRNAs may serve as excellent biomarkers for early detection of diseases.

miR-210 has been largely studied in the past several years and has been identified as a major hypoxia-induced miRNA which contributes to the induction of angiogenesis [9]. miR-210 can control cellular levels of VEGF through targeted regulation of receptor tyrosine kinase ligand Ephrin-A3 and phosphotyrosine phosphatase-1B [10]. The connective tissue growth factor (CTGF) has been shown to upregulate miR-210 expression to promote HIF-1*α*-dependent VEGF expression and angiogenesis in human synovial fibroblasts [11]. These findings provide evidence that miR-210 promotes angiogenesis.

In this study, we aimed to explore the clinical significance of miR-210 in synovial fluid samples from healthy volunteers and early-stage OA and late-stage OA patients. The results of our study shed new light on the identification of new diagnostic and prognostic markers for early detection of OA.

## 2. Materials and Methods

### 2.1. Specimens

The synovial fluid samples of normal and OA patients were obtained from the knee joints of patients undergoing arthrocentesis in the Luoyang Orthopedic Hospital of Henan Province and Third Affiliated Hospital of Xinxiang Medical College, China. The OA patients were divided into early- and late-stage groups according to Kellgren-Lawrence (KL) Grade: early stage (KL Grade: I/II) and late stage (KL Grade: III/IV). Written consent was obtained from all subjects prior to the recruitment and the study protocol was approved by the institutional review board of hospital ethics committee.

### 2.2. Quantification of miR-210

The synovial fluid sample was collected, immediately placed on ice and centrifuged at 3000 g for 10 min to remove cells and debris at 4°C. Supernatant was aliquoted and stored at -80°C until use. One ml of synovial fluid sample was thawed on ice and centrifuged at 3000 g for 5 min at 4°C before use. Total RNA containing miRNAs was extracted using isothiocyanate-phenol/chloroform extraction procedures. Real-time quantitative RT-PCR (qRT-PCR) was performed using SYBR® Premix DimerEraser kit (TaKaRa, Shiga, Japan) on an Bio-Rad CFX-96 real-time PCR system (Bio-Rad, Hercules, CA). U6 snRNA was used as an internal control. The primers for miR-210 and U6 were purchased from Guangzhou Ige Biotechnol (Guangzhou, China).

### 2.3. Quantification of VEGF by ELISA

The concentrations of VEGF in synovial fluid samples were measured by enzyme-linked immunosorbent assay (ELISA) in accordance with the manufacturer's recommendation (R&D Systems, Minneapolis, MN, USA).

### 2.4. Statistical Analysis

The difference in the expression of miRNAs among the groups was analyzed with Student's t-test. Logistic regression analyses were performed to evaluate the relationships between miR-210 and VEGF. All statistical analyses were done with GraphPad Prism 5 (La Jolla, CA, USA). P < 0.05 was considered significant.

## 3. Results

### 3.1. Clinical Characteristics of Study Subjects

Twenty early-stage OA patients, 20 late-stage OA patients, and 10 healthy age-matched individuals were recruited to this study. The average age at recruitment of normal control, early-stage OA, and late-stage OA patients was 63.8±8.5, 65.1±7.8, and 64.9±8.7 years, respectively. No significant difference in age and sex distribution was found among three study groups (P > 0.05) (Table 1).

### 3.2. miR-210 Expression Was Significantly Upregulated in Synovial Fluid Sample of Early-State and Late-Stage OA Patients

**Figure 1:**
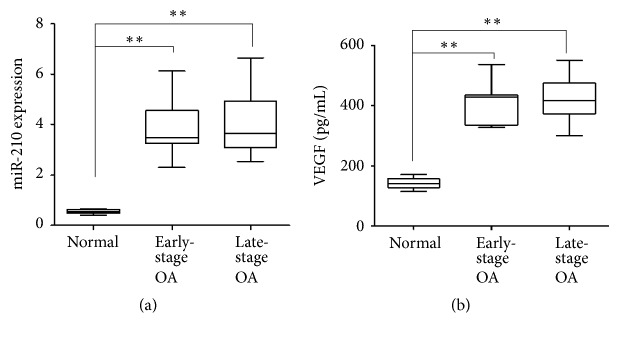
Expression of miR-210 and VEGF in normal, early-stage and late-stage OA patients. Synovial fluid samples were obtained from normal or OA patients. (a) miR-210 expression was measured by qRT-PCR. (b) VEGF was examined by ELISA. *∗∗* P < 0.01.

The expression levels of miR-210 were significantly higher in synovial fluid of early-stage and late-stage groups compared with the normal group (P < 0.01) (Figure 1(a)). No significant difference was found between miR-210 levels in synovial fluid of the early-stage and late-stage groups (Figure 1(a)).

### 3.3. Upregulation of VEGF Protein in OA Patients

Angiogenesis contributes to the OA synovial inflammation and is associated with disease severity. miR-210 has been reported to promote angiogenesis in cancer [12]. Therefore, we next investigated the protein expression of VEGF, a key factor in angiogenesis in OA [13]. We compared VEGF expression in synovial fluid samples in the three groups by ELISA. VEGF protein levels were significantly higher in early-stage and late-stage OA samples than in normal samples (P < 0.01) (Figure 1(b)). VEGF levels were similar in early- and late-stage OA samples (Figure 1(b)).

### 3.4. The Correlation of miR-210 with VEGF

**Figure 2:**
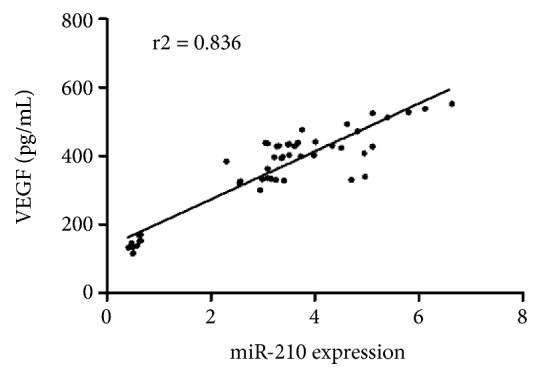
The correlation of miR-210 and VEGF in synovial fluid samples of normal and OA patients. Correlation was calculated in GraphPad Prism 5 as a measure of the degree of linear dependence between miR-210 and VEGF.

miR-210 expression is correlated closely with VEGF expression, hypoxia, and angiogenesis in breast cancer patients, indicating a possible role for miR-210 in tumor angiogenesis (19). Hence, the correlation study was used to assess association of miR-210 levels with VEGF expression, as seen in Figure 2, the R2 = 0.836 with a P < 0.01, indicating a positive correlation between miR-210 and VEGF.

## 4. Discussion

The limiting factors, such as an inability to early diagnose disease and a lack of understanding of the pathophysiology, result in ineffective OA therapeutics. Early diagnosis is vital for the treatment of OA. However, validated biomarkers for the early detection of OA remain to be identified.

The miRNAs, which can be detected in various body fluids including synovial fluid, have opened up new opportunities in discovering biomarkers in diseases [7]. Moreover, unlike distinct from miRNAs in plasma, miRNAs in synovial fluid were almost same as those secreted by synovial tissues due to their direct and intimate relationship with synovial membrane, articular cartilage, and other tissue types of knee joint, suggesting that synovial fluid miRNAs may be more suitable biomarkers for OA [14].

Several studies have assessed miRNA expression in synovial fluid samples of rheumatoid arthritis (RA) and OA patients. Murata K et al. investigated the presence of five miRNAs in OA synovial fluid and proved that miRNAs are present in synovial fluid samples in a stable form and can be a potential diagnostic marker for patients with RA and OA [14]. Li Y et al. screened over 750 miRNAs in synovial fluid from patients with early-stage and late-stage knee OA patients and identified a panel of seven circulating miRNAs that were significantly differentially expressed in synovial fluid samples from late-stage vs. early-stage OA patients [15]. Kolhe R et al. characterized exosomal miRNAs from synovial fluid of nonosteoarthritic and OA patients and concluded that synovial fluid exosomal miRNA content is altered with OA [16].

However, no similar miRNAs were reported from these studies and OA patients of our cohort. These differences might be due to limited patient number and disease stage. Li Y et al. determined miRNAs expression only in late-stage OA synovial fluid but not early-stage OA synovial fluid [15]. The synovial fluid samples in Kolhe's study [16] derived from patients with advanced OA, typically Grade 3 or 4, and the differentially expressed exosomal miRNAs may not be the same as those required for the diagnosis of early-stage OA.

Our study used synovial fluid samples derived from normal individual, early-stage, and late-stage OA patients and showed that, compared with normal control, synovial fluid miR-210 levels were significantly higher in OA patients, irrespective of stage, suggesting a potential of miR-210 as markers to early differentiate OA from suspect individuals. Our analysis further identified that miR-210 exhibited the significant association with VEGF, proving that enhanced angiogenesis may be a mechanism by which activation of miR-210 contributes to OA development. These results indicate that upregulation of miR-210 in synovial fluid may occur in the early stage of OA and can serve as a potential biomarker of early diagnosis in OA. Recent evidence has shown that miR-210 is also important in other type of arthritis. Yamasaki K et al. [17] reported that miR-210 was significantly higher in osteonecrosis compared to OA, suggesting that dysregulation of miR-210 is more associated with osteonecrosis. Abdul-Maksoud RS et al. [18] demonstrated that downregulation of serum miR-210 may be a novel biomarker for rheumatoid arthritis.

Whether miR-210 is involved in the progression of OA has not been fully illustrated. Recently, Li Z et al. [19] found that overexpression of miR-210 promoted chondrocyte proliferation and extracellular matrix deposition by targeting HIF-3*α* in OA. Zhang et al. [20] reported that MiR-210 inhibits NF-*κ*B signaling pathway by targeting DR6 in osteoarthritis. Further understanding the mechanism of miR-210 in OA may render miR-210 a promising target for OA treatment.

In conclusion, we have shown that miR-210 is significantly upregulated in synovial fluid samples of early-stage and late-stage OA patients and positively correlates with VEGF levels. These findings suggest that miR-210 in synovial fluid may have potential to be used as a noninvasive and rapid diagnostic tool for the prediction of susceptible individuals to developing OA. Further studies with a larger number of patients are warranted to validate these results.

## Figures and Tables

**Table 1:** Clinical characteristics of the study subjects.

Characteristics	Normal	Early-stage OA	Late-stage OA	P value
Sex				
Male	4	5	5	
Female	6	15	15	>0.05
Ages (yr)				
mean	63.8±8.5	65.1±7.8	64.9±8.7	
range	46-77	53-82	44-77	
median	65	65	67	>0.05

## Data Availability

The data used to support the findings of this study are included within the article.
